# Crystal structure of ethyl 2-(3,5-di­fluoro­phen­yl)quinoline-4-carboxyl­ate

**DOI:** 10.1107/S2056989015007677

**Published:** 2015-04-25

**Authors:** V. M. Sunitha, S. Naveen, H. R. Manjunath, S. B. Benaka Prasad, V. Manivannan, N. K. Lokanath

**Affiliations:** a Department of Physics, Shri Pillappa College of Engineering, Bengaluru 560 089, India; b Institution of Excellence, University of Mysore, Manasagangotri, Mysore 570 006, India; c Department of Physics, Acharya Institute of Technology, Soldevanahalli, Bengaluru 560 107, India; d Department of Chemistry, School of Engineering and Technology, Jain University, Bengaluru 562 012, India; e Department of Physics, Prist University, Vallam, Tanjavur 513 403, India; f Department of Studies in Physics, University of Mysore, Manasagangotri, Mysore 570 006, India

**Keywords:** crystal structure, quinoline derivatives, C—H⋯O hydrogen bonds

## Abstract

In the title compound, C_18_H_13_F_2_NO_2_, the two rings of the quinoline system are fused almost coaxially, with a dihedral angle between their planes of 2.28 (8)°. The plane of the attached benzene ring is inclined to the plane of the quinoline system by 7.65 (7)°. The carboxyl­ate group attached to the quinoline system is in an anti­periplanar conformation. There is a short intra­molecular C—H⋯O contact involving the carbonyl group. In the crystal, mol­ecules are linked *via* C—H⋯O hydrogen bonds, forming chains lying in the (1-10) plane.

## Related literature   

For the crystal structures of related quinoline derivatives, see: Pradeep *et al.* (2014 ▸); Shrungesh Kumar *et al.* (2015 ▸).
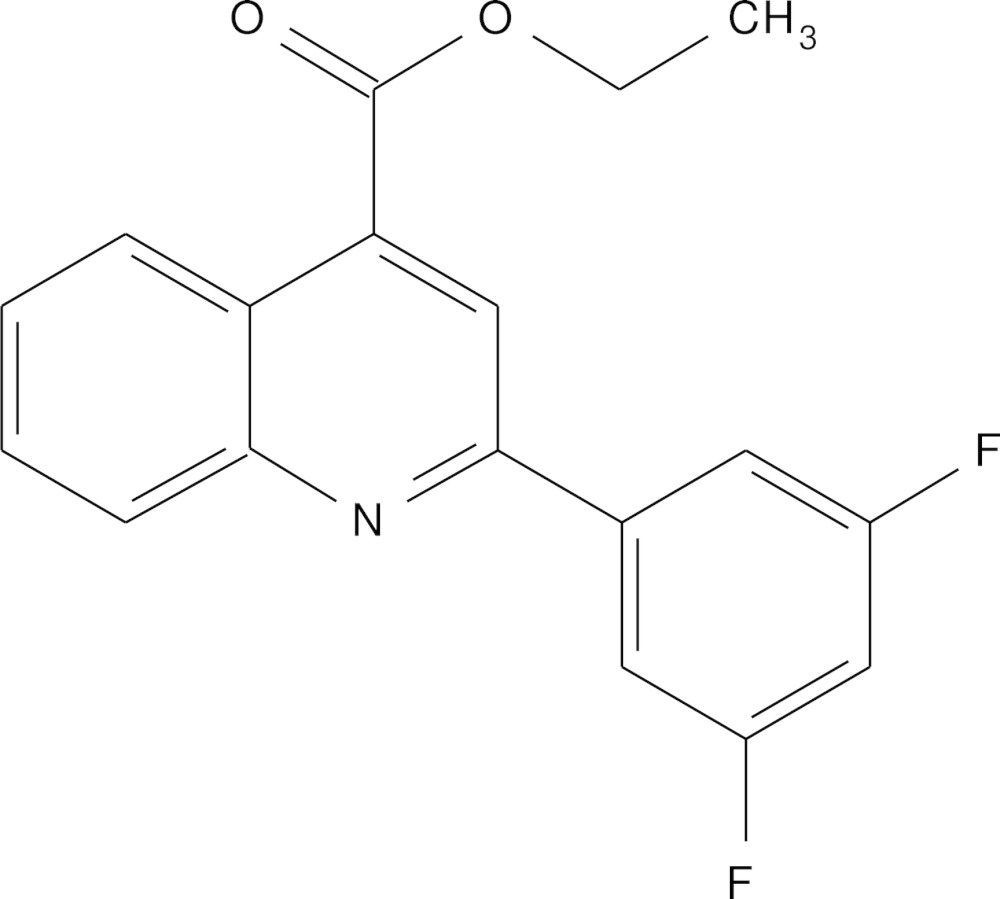


## Experimental   

### Crystal data  

C_18_H_13_F_2_NO_2_
*M* *_r_* = 313.29
Triclinic, 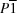

*a* = 8.2674 (3) Å
*b* = 10.0529 (4) Å
*c* = 10.0562 (4) Å
α = 101.193 (2)°
β = 108.616 (2)°
γ = 98.741 (2)°
*V* = 756.14 (5) Å^3^
*Z* = 2
Cu *K*α radiation
μ = 0.90 mm^−1^
*T* = 296 K
0.30 × 0.27 × 0.25 mm

### Data collection  

Bruker X8 Proteum diffractometer
Absorption correction: multi-scan (*SADABS*; Bruker, 2013 ▸) *T*_min_ = 0.763, *T*_max_ = 0.799
9032 measured reflections
2482 independent reflections
2147 reflections with *I* > 2σ(*I*)
*R*_int_ = 0.038

### Refinement  

*R*[*F* ^2^ > 2σ(*F* ^2^)] = 0.044
*wR*(*F* ^2^) = 0.131
*S* = 1.05
2482 reflections
210 parameters
H-atom parameters constrained
Δρ_max_ = 0.16 e Å^−3^
Δρ_min_ = −0.22 e Å^−3^

### 

Data collection: *APEX2* (Bruker, 2013 ▸); cell refinement: *SAINT* (Bruker, 2013 ▸); data reduction: *SAINT*; program(s) used to solve structure: *SHELXS97* (Sheldrick, 2008 ▸); program(s) used to refine structure: *SHELXL97* (Sheldrick, 2008 ▸); molecular graphics: *Mercury* (Macrae *et al.*, 2008 ▸); software used to prepare material for publication: *SHELXL97*.

## Supplementary Material

Crystal structure: contains datablock(s) global, I. DOI: 10.1107/S2056989015007677/su5120sup1.cif

Structure factors: contains datablock(s) I. DOI: 10.1107/S2056989015007677/su5120Isup2.hkl

Supporting information file. DOI: 10.1107/S2056989015007677/su5120Isup3.cml

**Fig. 1.**
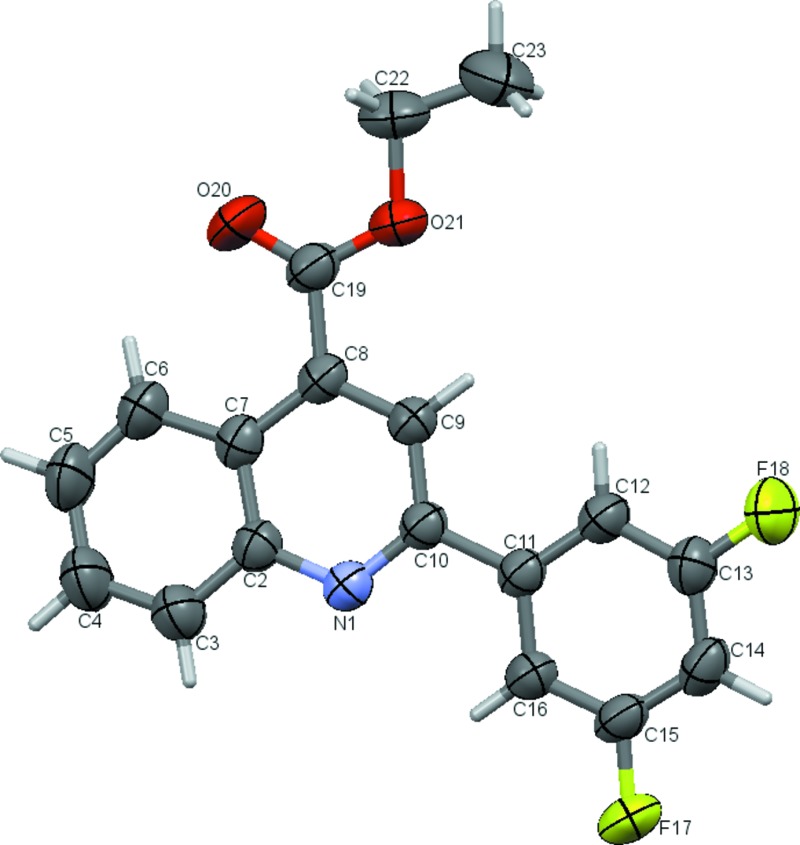
View of the molecular structure of the title compound, with atom labelling. Displacement ellipsoids are drawn at the 50% probability level.

**Fig. 2.**
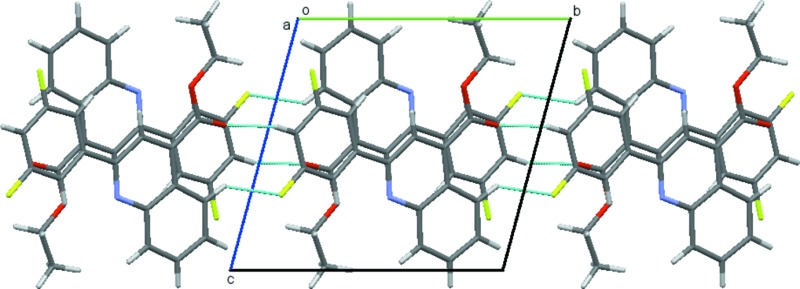
A view along the a axis of the crystal packing of the title compound. Hydrogen bonds are shown as dashed lines (see Table 1 for details).

. DOI: 10.1107/S2056989015007677/su5120fig1.tif
View of the mol­ecular structure of the title compound, with atom labelling. Displacement ellipsoids are drawn at the 50% probability level.

a . DOI: 10.1107/S2056989015007677/su5120fig2.tif
A view along the *a* axis of the crystal packing of the title compound. Hydrogen bonds are shown as dashed lines (see Table 1 for details).

CCDC reference: 1060299

Additional supporting information:  crystallographic information; 3D view; checkCIF report

## Figures and Tables

**Table 1:** Hydrogen-bond geometry (, )

*D*H*A*	*D*H	H*A*	*D* *A*	*D*H*A*
C6H6O20	0.93	2.24	2.861(2)	123
C14H14O20^i^	0.93	2.36	3.275(2)	167

Symmetry code: (i) 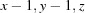.

## supplementary crystallographic information

### S1. Comment

Quinoline derivatives are well known for their wide range of biological and pharmaceutical activities and are prevalent in pharmacologically active natural and synthetic compounds. Quinoline-4-carboxyl­ates are potential 5HT_3_ antagonizer and also they possess anti-emetic activity. In view of their broad spectrum of medicinal properties and in continuation of our work on new quinoline-based therapeutic agents (Pradeep *et al.*, 2014, Shrungesh Kumar *et al.*, 2015), we have synthesized the title compound and report herein on its crystal structure.

The structure of the compound is shown in Fig. 1. The dihedral angle between the benzene ring and the quinoline ring system is 7.65 (7) °. The carboxyl­ate group attached to the quinoline moiety is in an *-anti periplanar* conformation which is evident by the torsion angle C22—O21—C19—C8 = -176.71 (15)°. The two rings of the quinoline moiety are fused in an axial fashion with a dihedral angle value of 2.28 (8)°. The deviation of the bond length values for C8—C19 and C10—C11 from the standard values can be attributed for the *sp^3^* hybridization.

In the crystal, molecules are linked via C—H···O hydrogen bonds forming chains lying in the (110) plane. (Fig. 2 and Table 1).

### S2. Synthesis and crystallization

To a solution of 2-(3,5-di­fluoro­phenyl)­quinoline-4-carb­oxy­lic acid (0.5 g) in 20 ml of EtOH, 1 ml of H_2_SO_4_ (conc.) was added. The resulting reaction mixture was refluxed for 15 h. Solvent was removed under reduced pressure and the residue was partitioned between EtOAc and saturated NaHCO_3_ solution. The organic layer was washed with water and brine, dried over anhydrous Na_2_SO_4_, filtered, and condensed to give the title compound as a white solid (yield: 93%). The compound was dissolved in di­methyl­formamide and the solution was gently heated and left undisturbed. Brown, re­cta­ngular crystals grew after 12 days by slow evaporation of the solvent.

### S3. Refinement

Crystal data, data collection and structure refinement details are summarized in Table 2. All the H atoms were fixed geometrically (C—H= 0.93–0.96 Å) and allowed to ride on their parent atoms with *U*_iso_(H) = 1.5*U*_eq_(C) for methyl H atoms and 1.2*U*_eq_(C) for other H atoms.

### Crystal data

**Table:**

C_18_H_13_F_2_NO_2_	*Z* = 2
*M**_r_* = 313.29	*F*(000) = 324
Triclinic, *P*1	*D*_x_ = 1.376 Mg m^−^^3^
Hall symbol: -P 1	Cu *K*α radiation, λ = 1.54178 Å
*a* = 8.2674 (3) Å	Cell parameters from 2482 reflections
*b* = 10.0529 (4) Å	θ = 6.1–64.4°
*c* = 10.0562 (4) Å	µ = 0.90 mm^−^^1^
α = 101.193 (2)°	*T* = 296 K
β = 108.616 (2)°	Rectangle, brown
γ = 98.741 (2)°	0.30 × 0.27 × 0.25 mm
*V* = 756.14 (5) Å^3^	

### Data collection

**Table:**

Bruker X8 Proteum diffractometer	2482 independent reflections
Radiation source: Bruker MicroStar microfocus rotating anode	2147 reflections with *I* > 2σ(*I*)
Helios multilayer optics monochromator	*R*_int_ = 0.038
Detector resolution: 10.7 pixels mm^-1^	θ_max_ = 64.4°, θ_min_ = 6.1°
φ and ω scans	*h* = −9→9
Absorption correction: multi-scan (*SADABS*; Bruker, 2013)	*k* = −11→11
*T*_min_ = 0.763, *T*_max_ = 0.799	*l* = −11→11
9032 measured reflections	

### Refinement

**Table:**

Refinement on *F*^2^	Secondary atom site location: difference Fourier map
Least-squares matrix: full	Hydrogen site location: inferred from neighbouring sites
*R*[*F*^2^ > 2σ(*F*^2^)] = 0.044	H-atom parameters constrained
*wR*(*F*^2^) = 0.131	*w* = 1/[σ^2^(*F*_o_^2^) + (0.0696*P*)^2^ + 0.099*P*] where *P* = (*F*_o_^2^ + 2*F*_c_^2^)/3
*S* = 1.05	(Δ/σ)_max_ < 0.001
2482 reflections	Δρ_max_ = 0.16 e Å^−^^3^
210 parameters	Δρ_min_ = −0.22 e Å^−^^3^
0 restraints	Extinction correction: *SHELXL97* (Sheldrick, 2008), FC^*^ = KFC[1+0.001XFC^2^Λ^3^/SIN(2Θ)]^-1/4^
Primary atom site location: structure-invariant direct methods	Extinction coefficient: 0.011 (3)

### Special details

**Table:**

Geometry. Bond distances, angles *etc*. have been calculated using the rounded fractional coordinates. All su's are estimated from the variances of the (full) variance-covariance matrix. The cell e.s.d.'s are taken into account in the estimation of distances, angles and torsion angles
Refinement. Refinement on *F*^2^ for ALL reflections except those flagged by the user for potential systematic errors. Weighted *R*-factors *wR* and all goodnesses of fit *S* are based on *F*^2^, conventional *R*-factors *R* are based on *F*, with *F* set to zero for negative *F*^2^. The observed criterion of *F*^2^ > σ(*F*^2^) is used only for calculating -*R*-factor-obs *etc*. and is not relevant to the choice of reflections for refinement. *R*-factors based on *F*^2^ are statistically about twice as large as those based on *F*, and *R*-factors based on ALL data will be even larger.

### Fractional atomic coordinates and isotropic or equivalent isotropic displacement parameters (Å^2^)

**Table:**

	*x*	*y*	*z*	*U*_iso_*/*U*_eq_	
F17	−0.07505 (13)	0.11215 (10)	0.69978 (11)	0.0832 (4)	
F18	−0.21878 (16)	0.10400 (12)	0.20982 (12)	0.0993 (4)	
O20	0.5498 (2)	0.83249 (15)	0.42261 (16)	0.1012 (6)	
O21	0.31565 (15)	0.67459 (11)	0.26915 (12)	0.0665 (4)	
N1	0.35974 (15)	0.51312 (12)	0.70955 (13)	0.0525 (4)	
C2	0.48895 (19)	0.63200 (14)	0.75527 (16)	0.0520 (4)	
C3	0.5941 (2)	0.67423 (17)	0.90376 (18)	0.0698 (6)	
C4	0.7210 (3)	0.7948 (2)	0.9594 (2)	0.0830 (7)	
C5	0.7486 (3)	0.87609 (19)	0.8681 (2)	0.0837 (7)	
C6	0.6535 (2)	0.83789 (16)	0.72399 (19)	0.0682 (6)	
C7	0.51962 (18)	0.71364 (14)	0.66138 (16)	0.0512 (5)	
C8	0.41001 (18)	0.66272 (14)	0.51179 (16)	0.0477 (4)	
C9	0.28096 (18)	0.54385 (14)	0.46942 (15)	0.0475 (4)	
C10	0.25718 (17)	0.47149 (13)	0.57158 (15)	0.0458 (4)	
C11	0.11409 (17)	0.34387 (13)	0.52808 (15)	0.0473 (4)	
C12	0.0105 (2)	0.28135 (15)	0.38426 (17)	0.0578 (5)	
C13	−0.1193 (2)	0.16400 (16)	0.35114 (17)	0.0622 (5)	
C14	−0.1529 (2)	0.10394 (15)	0.45354 (19)	0.0610 (5)	
C15	−0.0474 (2)	0.16870 (15)	0.59420 (18)	0.0577 (5)	
C16	0.08370 (19)	0.28567 (15)	0.63472 (17)	0.0544 (5)	
C19	0.4343 (2)	0.73427 (15)	0.39981 (18)	0.0573 (5)	
C22	0.3365 (3)	0.7336 (2)	0.1529 (2)	0.0850 (8)	
C23	0.1898 (4)	0.6564 (3)	0.0175 (3)	0.1138 (10)	
H3	0.57680	0.61930	0.96470	0.0840*	
H4	0.78890	0.82250	1.05790	0.1000*	
H5	0.83450	0.95880	0.90700	0.1000*	
H6	0.67630	0.89370	0.66540	0.0820*	
H9	0.20810	0.51030	0.37230	0.0570*	
H12	0.02840	0.31830	0.31080	0.0690*	
H14	−0.24150	0.02450	0.42900	0.0730*	
H16	0.15150	0.32560	0.73220	0.0650*	
H22A	0.44750	0.72490	0.14270	0.1020*	
H22B	0.33500	0.83160	0.17440	0.1020*	
H23A	0.18670	0.55860	0.00140	0.1710*	
H23B	0.20580	0.68790	−0.06260	0.1710*	
H23C	0.08140	0.67240	0.02550	0.1710*	

### Atomic displacement parameters (Å^2^)

**Table:**

	*U*^11^	*U*^22^	*U*^33^	*U*^12^	*U*^13^	*U*^23^
F17	0.0858 (7)	0.0796 (7)	0.0865 (7)	−0.0067 (5)	0.0321 (6)	0.0446 (5)
F18	0.1077 (9)	0.0813 (7)	0.0655 (7)	−0.0309 (6)	0.0073 (6)	0.0027 (5)
O20	0.1060 (11)	0.0881 (9)	0.0877 (10)	−0.0363 (8)	0.0244 (8)	0.0372 (7)
O21	0.0731 (7)	0.0690 (7)	0.0589 (7)	0.0017 (5)	0.0253 (6)	0.0284 (5)
N1	0.0544 (7)	0.0478 (7)	0.0520 (7)	0.0038 (5)	0.0171 (6)	0.0154 (5)
C2	0.0507 (8)	0.0465 (7)	0.0545 (8)	0.0059 (6)	0.0162 (7)	0.0122 (6)
C3	0.0755 (11)	0.0633 (10)	0.0568 (9)	0.0024 (8)	0.0119 (8)	0.0151 (7)
C4	0.0820 (12)	0.0736 (11)	0.0620 (11)	−0.0057 (9)	0.0003 (9)	0.0091 (9)
C5	0.0757 (12)	0.0615 (10)	0.0820 (13)	−0.0157 (8)	0.0065 (10)	0.0086 (9)
C6	0.0654 (10)	0.0546 (9)	0.0720 (11)	−0.0056 (7)	0.0173 (8)	0.0156 (8)
C7	0.0478 (8)	0.0445 (7)	0.0587 (9)	0.0066 (6)	0.0186 (7)	0.0118 (6)
C8	0.0463 (7)	0.0436 (7)	0.0572 (8)	0.0086 (6)	0.0225 (6)	0.0168 (6)
C9	0.0464 (7)	0.0455 (7)	0.0497 (8)	0.0064 (6)	0.0166 (6)	0.0145 (6)
C10	0.0452 (7)	0.0428 (7)	0.0503 (8)	0.0082 (6)	0.0178 (6)	0.0145 (6)
C11	0.0454 (7)	0.0431 (7)	0.0547 (8)	0.0080 (6)	0.0187 (6)	0.0163 (6)
C12	0.0628 (9)	0.0505 (8)	0.0564 (9)	0.0002 (7)	0.0208 (7)	0.0169 (7)
C13	0.0615 (9)	0.0513 (8)	0.0595 (9)	−0.0003 (7)	0.0129 (7)	0.0079 (7)
C14	0.0540 (9)	0.0432 (8)	0.0819 (11)	0.0004 (6)	0.0227 (8)	0.0185 (7)
C15	0.0561 (9)	0.0513 (8)	0.0731 (10)	0.0075 (6)	0.0270 (8)	0.0303 (7)
C16	0.0525 (8)	0.0534 (8)	0.0567 (9)	0.0053 (6)	0.0175 (7)	0.0217 (7)
C19	0.0578 (9)	0.0518 (8)	0.0661 (10)	0.0065 (7)	0.0258 (8)	0.0227 (7)
C22	0.0999 (14)	0.0970 (14)	0.0718 (12)	0.0123 (11)	0.0406 (11)	0.0442 (10)
C23	0.1130 (18)	0.156 (2)	0.0710 (13)	0.0141 (16)	0.0238 (12)	0.0552 (14)

### Geometric parameters (Å, º)

**Table:**

F17—C15	1.3611 (19)	C11—C16	1.388 (2)
F18—C13	1.3528 (19)	C12—C13	1.374 (2)
O20—C19	1.194 (2)	C13—C14	1.369 (2)
O21—C19	1.319 (2)	C14—C15	1.367 (2)
O21—C22	1.454 (2)	C15—C16	1.367 (2)
N1—C2	1.366 (2)	C22—C23	1.475 (4)
N1—C10	1.3171 (18)	C3—H3	0.9300
C2—C3	1.407 (2)	C4—H4	0.9300
C2—C7	1.421 (2)	C5—H5	0.9300
C3—C4	1.363 (3)	C6—H6	0.9300
C4—C5	1.390 (3)	C9—H9	0.9300
C5—C6	1.353 (3)	C12—H12	0.9300
C6—C7	1.418 (2)	C14—H14	0.9300
C7—C8	1.427 (2)	C16—H16	0.9300
C8—C9	1.368 (2)	C22—H22A	0.9700
C8—C19	1.497 (2)	C22—H22B	0.9700
C9—C10	1.414 (2)	C23—H23A	0.9600
C10—C11	1.492 (2)	C23—H23B	0.9600
C11—C12	1.383 (2)	C23—H23C	0.9600
			
C19—O21—C22	115.85 (14)	O20—C19—O21	122.45 (16)
C2—N1—C10	118.74 (12)	O20—C19—C8	124.87 (16)
N1—C2—C3	117.08 (14)	O21—C19—C8	112.65 (14)
N1—C2—C7	123.41 (13)	O21—C22—C23	107.77 (19)
C3—C2—C7	119.51 (14)	C2—C3—H3	120.00
C2—C3—C4	120.79 (16)	C4—C3—H3	120.00
C3—C4—C5	119.67 (17)	C3—C4—H4	120.00
C4—C5—C6	121.59 (19)	C5—C4—H4	120.00
C5—C6—C7	120.72 (16)	C4—C5—H5	119.00
C2—C7—C6	117.68 (14)	C6—C5—H5	119.00
C2—C7—C8	116.27 (13)	C5—C6—H6	120.00
C6—C7—C8	126.04 (14)	C7—C6—H6	120.00
C7—C8—C9	118.95 (13)	C8—C9—H9	120.00
C7—C8—C19	121.86 (13)	C10—C9—H9	120.00
C9—C8—C19	119.19 (13)	C11—C12—H12	120.00
C8—C9—C10	120.74 (13)	C13—C12—H12	120.00
N1—C10—C9	121.83 (13)	C13—C14—H14	122.00
N1—C10—C11	116.69 (12)	C15—C14—H14	122.00
C9—C10—C11	121.48 (12)	C11—C16—H16	121.00
C10—C11—C12	121.97 (13)	C15—C16—H16	121.00
C10—C11—C16	119.24 (13)	O21—C22—H22A	110.00
C12—C11—C16	118.79 (14)	O21—C22—H22B	110.00
C11—C12—C13	119.22 (14)	C23—C22—H22A	110.00
F18—C13—C12	118.35 (14)	C23—C22—H22B	110.00
F18—C13—C14	118.13 (15)	H22A—C22—H22B	108.00
C12—C13—C14	123.52 (15)	C22—C23—H23A	109.00
C13—C14—C15	115.41 (15)	C22—C23—H23B	109.00
F17—C15—C14	117.53 (14)	C22—C23—H23C	110.00
F17—C15—C16	118.38 (14)	H23A—C23—H23B	109.00
C14—C15—C16	124.09 (15)	H23A—C23—H23C	109.00
C11—C16—C15	118.97 (14)	H23B—C23—H23C	109.00
			
C19—O21—C22—C23	−178.32 (19)	C19—C8—C9—C10	178.34 (14)
C22—O21—C19—O20	1.5 (3)	C9—C8—C19—O21	3.7 (2)
C22—O21—C19—C8	−176.71 (15)	C7—C8—C19—O20	4.4 (3)
C10—N1—C2—C7	−0.3 (2)	C7—C8—C19—O21	−177.37 (14)
C10—N1—C2—C3	179.08 (15)	C9—C8—C19—O20	−174.49 (18)
C2—N1—C10—C9	2.1 (2)	C8—C9—C10—N1	−1.7 (2)
C2—N1—C10—C11	−177.91 (13)	C8—C9—C10—C11	178.32 (14)
N1—C2—C3—C4	−177.18 (18)	N1—C10—C11—C12	−172.73 (14)
C3—C2—C7—C6	−1.9 (2)	C9—C10—C11—C16	−172.80 (14)
C3—C2—C7—C8	178.80 (15)	N1—C10—C11—C16	7.2 (2)
N1—C2—C7—C6	177.45 (15)	C9—C10—C11—C12	7.3 (2)
N1—C2—C7—C8	−1.9 (2)	C16—C11—C12—C13	0.1 (2)
C7—C2—C3—C4	2.2 (3)	C10—C11—C12—C13	−179.96 (16)
C2—C3—C4—C5	−0.9 (3)	C10—C11—C16—C15	−179.93 (15)
C3—C4—C5—C6	−0.7 (4)	C12—C11—C16—C15	0.0 (2)
C4—C5—C6—C7	1.0 (3)	C11—C12—C13—F18	−179.73 (15)
C5—C6—C7—C2	0.3 (3)	C11—C12—C13—C14	−0.1 (3)
C5—C6—C7—C8	179.57 (18)	C12—C13—C14—C15	−0.1 (3)
C6—C7—C8—C9	−177.03 (16)	F18—C13—C14—C15	179.58 (15)
C2—C7—C8—C19	−176.71 (14)	C13—C14—C15—F17	−179.53 (15)
C2—C7—C8—C9	2.2 (2)	C13—C14—C15—C16	0.2 (3)
C6—C7—C8—C19	4.0 (3)	F17—C15—C16—C11	179.57 (14)
C7—C8—C9—C10	−0.6 (2)	C14—C15—C16—C11	−0.2 (3)

### Hydrogen-bond geometry (Å, º)

**Table:**

*D*—H···*A*	*D*—H	H···*A*	*D*···*A*	*D*—H···*A*
C6—H6···O20	0.93	2.24	2.861 (2)	123
C14—H14···O20^i^	0.93	2.36	3.275 (2)	167

Symmetry code: (i) *x*−1, *y*−1, *z*.
