# Supplementary figures and images for: Crystal structure of ethyl 2-(3,5-di­fluoro­phen­yl)quinoline-4-carboxyl­ate
Source: Acta Crystallogr E Crystallogr Commun. 2015 Apr 25;71(Pt 5):o341–2. doi: 10.1107/S2056989015007677 (PMC4420057; doi:10.1107/S2056989015007677)

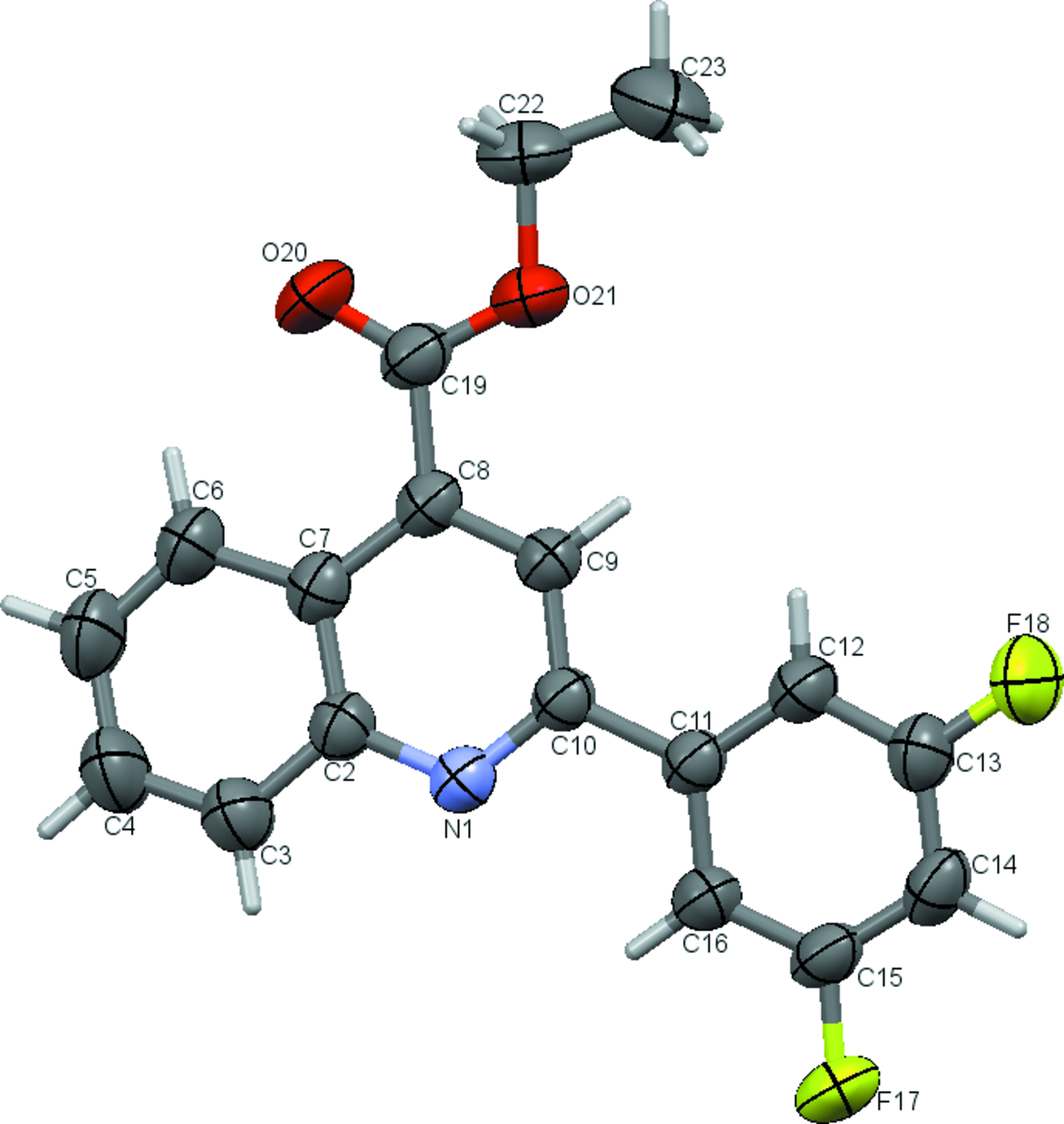

Supplement: Supplementary file 4 [file e-71-0o341-fig1.tif]

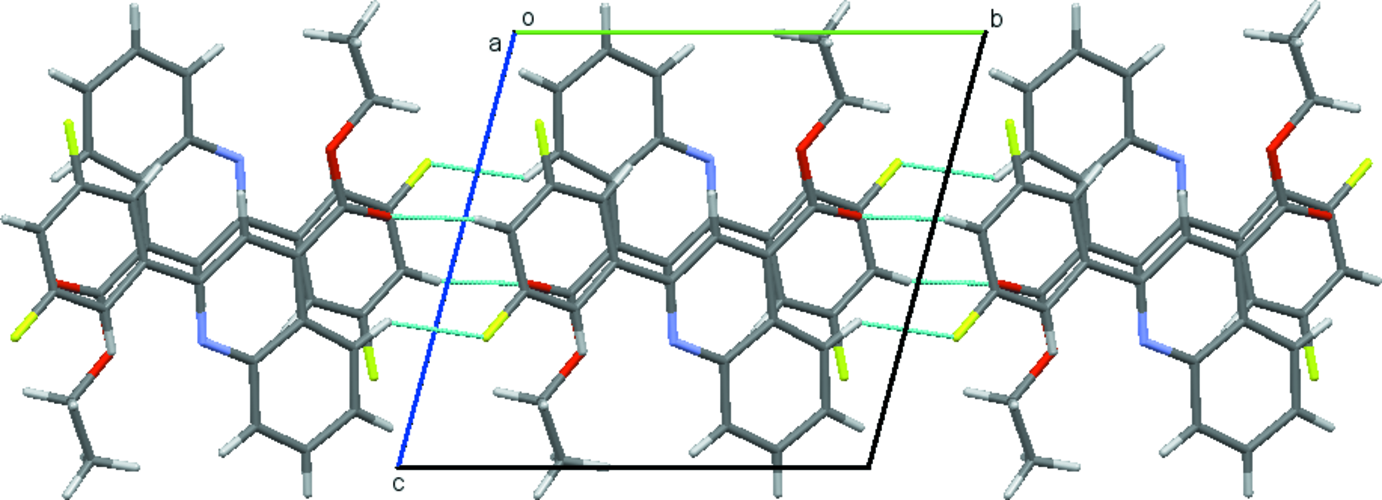

Supplement: Supplementary file 5 [file e-71-0o341-fig2.tif]
